# An Uncommon Cause of Urinary Tract Infections: A Case Report

**DOI:** 10.7759/cureus.23325

**Published:** 2022-03-19

**Authors:** Jonathan Otero-Colón, Kristen L Farraj, Zalak Desai

**Affiliations:** 1 Internal Medicine, Nassau University Medical Center, East Meadow, USA

**Keywords:** aerococcus urinae, aerococcus, bacteremia, sepsis, urinary tract infection

## Abstract

Urinary tract infections (UTI) in the elderly are common. UTI ranges in severity from mild disease to severe sepsis. Many organisms can cause UTIs yet many UTIs are caused by the same few organisms. An organism that has been increasingly gaining notoriety for infections is Aerococcus urinae. Aerococcus infections are constantly misdiagnosed due to their difficulty to identify. Here we present a case of an elderly male who was found to have a urinary tract infection with Aerococcus urinaethat progressed into bacteremia, severe sepsis and ultimately death.

## Introduction

In the United States, there were approximately 10.50 million ambulatory visits and 2-3 million emergency department visits for urinary tract infections (UTIs) in 2007, and $3.50 billion were spent in 2015 [1]. To understand the magnitude of how such simple infections can take a toll on the population of medical care we must understand these infections. UTI is defined as a kidney infection that can lead to systemic symptoms such as fever and weakness and can cause discomfort and difficulty with daily activities [1]. UTI ranges in severity from mild self-limitation to severe sepsis, with a mortality rate of 20-40% [1]. Specifically, in this case we will discuss a common UTI in an elderly male which was caused by an uncommon organism. UTIs in males are less common than females and are commonly accompanied by coexisting conditions that facilitate such infections. Benign prostatic hyperplasia (BPH) is defined as a benign proliferation of prostatic tissue, often leading to symptoms such as urinary retention, bladder outlet obstruction, or urinary tract infections [1].

Many organisms have the potential to cause UTIs although many UTIs are caused by a few organisms. An organism that has been increasingly gaining notoriety for infections is Aerococcus urinae (A. urinae). Aerococcus infections are constantly misdiagnosed due to their difficulty to identify. First reported in 1967, the organisms belonged to a loosely associated bacterial group referred to as Aerococcus-like organisms (ALO). This group previously found in breweries, meat-curing brines, lobsters, and horse urine caused opportunistic endocarditis and urinary tract infections [2]. Understanding the organism provides insight into understanding the infection. Aerococci appear as pairs, tetrads or clusters in the Gram stain, but unlike staphylococci they do not produce catalase. On blood agar plates, they are alpha haemolytic and resemble streptococci, displaying small semi-transparent colonies. Growth occurs both under aerobic and anaerobic conditions [3]. Concerning the impact to clinicians is the control and eradication of such infections. The sensibilities and susceptibilities to widely used antibiotics is paramount. Most aerococci are sensitive to beta-lactam antibiotics as well as to several other groups of antibiotics. The combination of penicillin and netilmicin or gentamicin has been demonstrated to have a synergistic killing effect on two A. urinae endocarditis isolates in vitro. Aerococcus spp are sensitive to vancomycin, tetracycline, erythromycin, clindamycin, and rifampicin [3]. Here we present a case of a 93-year-old Caucasian male who was found to have bacteremia secondary to a urinary tract infection from Aerococcus urinae.

## Case presentation

A 93-year-old Caucasian male with a past history of type 2 diabetes mellitus and benign prostatic hyperplasia presented to ED due to a fall outside of his home that resulted in head and arm trauma. As per the patient, he lived alone and had cured his diabetes and was only taking multivitamins at home. He was subsequently admitted for further evaluation and as per the patient's daughter, the patient had become increasingly confused. Radiological evaluation including computed tomography (CT) head, CT cervical spine, CT abdomen and pelvis, and CT maxillofacial demonstrated no acute injury with exception to radial fracture. The orthopedic team was consulted and a closed reduction of his radial fracture was performed, which the patient tolerated well. On admission labs, his creatinine was elevated at 1.5 mg/dL with an unknown baseline. It was an unclear etiology of acute kidney injury superimposed on chronic kidney injury. On day 2 of admission, the patient became confused and agitated in the setting of acute urinary retention. A foley catheter was placed and was positive for gross hematuria. Urinalysis was sent which was positive for macroscopic hematuria and few bacteria. Following the insertion of the foley catheter, the patient's acute kidney injury resolved. On the third day of hospitalization, the patient developed leukocytosis but remained afebrile. At this time, urine culture results had come back positive for Aerococcus urinae. Blood cultures were also drawn and were positive for bacteremia in two out of four bottles with gram-positive cocci in clusters, which was found to be Aerococcus urinae. Broad-spectrum antibiotics were initiated with ceftriaxone and vancomycin, however, he developed a rash after vancomycin administration and it was discontinued. He was then placed on clindamycin but due to the persistent and rising leukocytosis with a peak of 23.18 k/mm³ along with sporadic fevers, ampicillin/sulbactam was also added. An echocardiogram was performed to rule out endocarditis in the setting of bacteremia and was found to be negative for any vegetations. Despite repeated blood cultures demonstrating no bacterial growth after five days and completing the antibiotic courses for both ceftriaxone and clindamycin, he was still febrile with a maximum temperature of 101.3 degrees Fahrenheit. Linezolid was then added along with ampicillin/sulbactam the patient was on. He subsequently completed the course of both antibiotics but yet still had leukocytosis and throughout his stay, his PO intake gradually decreased. This only increased his severe malnourishment and he continued to be septic with tachycardia and hypotension despite appropriate management. The nutrition team was consulted and recommendations were followed for additional supplements with meals. Goals of care were discussed with the patient's daughter who decided her father would have chosen comfort measures at this time. All medical interventions were discontinued except for morphine and dronabinol in an effort to keep the patient as comfortable as possible and stimulate appetite. Arrangements were made for the patient to be transferred to outpatient hospice care, but the patient expired prior to discharge. The probable cause of death at this time was failure to thrive and severe malnutrition despite all efforts.

## Discussion

As Aerococcus urinae continues to become a well-known invasive organism causing infection and disease, various obstacles remain present. Aerococcus urinae was first recognized as a separate species in 1992, yet its normal habitat remains unclear. Interestingly, it shares characteristics with Streptococci, Staphylococci, and Enterococci [4]. These similarities have made it challenging to appropriately identify and therefore treat infections with increasing specificity. Aerococcus urinae exhibits alpha hemolysis on blood agar, grows in clusters, and is intrinsically resistant to sulfonamide. Unlike Staphylococcus, it does not produce catalase. Its growth occurs under both aerobic and anaerobic conditions [4]. Utilizing these differences facilitates the identification and subsequent management of this invasive species.

The need for prompt identification and treatment increases as newer sequelae of disease are discovered. Rare reports refer to A. urinae as a cause of invasive human infections, such as sepsis, endocarditis, peritonitis, spondylodiscitis and vertebral osteomyelitis [5]. Unidentified cases can rapidly deteriorate into major infections as in the case of necrotizing urethritis. Necrotizing soft tissue infections are fulminant infections affecting the soft tissue compartment and leading to widespread necrosis, systemic toxicity, and a high mortality rate if not treated early [4]. Babaeer et al. discussed a severe Aerococcus urinae urinary tract infection that resulted in necrotizing urethritis in a relatively young man [4]. Although A. urinae commonly leads to genitourinary tract infections cases of other organs being involved are emerging. For example, Yabes et al. reported a case of infective endocarditis secondary to ​​​​​​A. urinae [6]. To date, the overall incidence of infective endocarditis due to A. urinae is unknown, but with increasingly sophisticated laboratory techniques the reported incidence of A. urinae is increasing [6]. Due to the possibility of severe complications, it is imperative that risk factors are identified. Recognized risk factors for invasive A. urinae infection include male gender, age greater than 65 years, and pre-existing urinary tract pathology [6]. In this case, our patient was an elderly male with an age greater than 65 with BPH.

Once suspicion is encountered, targeted treatment is of utmost importance. Due to known sensitivities, our patient discussed above was started on vancomycin and ceftriaxone with vancomycin exchanged for clindamycin due to delayed allergic reaction. Despite these antibiotics, the patient still had febrile episodes. In comparison, Jerome et al. reported a case where the patient experienced clinical improvement after six weeks of cefazolin [7]. Due to microbial resistance, our patient was started on a second course of antibiotics with ampicillin/sulbactam and linezolid which demonstrated resolution of the current infection. Aerococcus urinae is not known to be especially resistant. Resistance in this case could have been due to biofilm formation. Fastidious microbes tolerating oxygen (A. urinae and G. sanguinis) were identified in catheter biofilms, but less common compared to the genetically related E. faecalis [8]. Unfortunately, due to other comorbidities the patient expired.

## Conclusions

Aerococcus urinae is an emerging invasive organism. The range and degree to which infections can lead to disease has not been quantified. Although common infections with A. urinae lead to minor UTIs, the possibility for complications is becoming even more apparent. In this case, an elderly male presented with a minor infection that through antimicrobial resistance led to recalcitrant disease that required a range of antibiotics. Throughout literature, infections with A. urinae are gaining notice for their complications that lead from severe UTIs to seeding other tissues including bone and cardiac muscle. For these reasons, the identification and prompt treatment of A. urinae is ever more paramount. Lastly, careful consideration must be taken in regards to antibiotic choice with resistant strains as to not propagate further resistance.
